# Circadian Regulation of Apolipoproteins in the Brain: Implications in Lipid Metabolism and Disease

**DOI:** 10.3390/ijms242417415

**Published:** 2023-12-12

**Authors:** Chaeeun Hannah Lee, Charlotte Ellzabeth Murrell, Alexander Chu, Xiaoyue Pan

**Affiliations:** 1 Department of Foundations of Medicine, New York University Grossman Long Island School of Medicine, Mineola, NY 11501, USA; 2 Diabetes and Obesity Research Center, NYU Langone Hospital-Long Island, Mineola, NY 11501, USA

**Keywords:** lipid, apolipoprotein, circadian clock, hormone, neuron, brain

## Abstract

The circadian rhythm is a 24 h internal clock within the body that regulates various factors, including sleep, body temperature, and hormone secretion. Circadian rhythm disruption is an important risk factor for many diseases including neurodegenerative illnesses. The central and peripheral oscillators’ circadian clock network controls the circadian rhythm in mammals. The clock genes govern the central clock in the suprachiasmatic nucleus (SCN) of the brain. One function of the circadian clock is regulating lipid metabolism. However, investigations of the circadian regulation of lipid metabolism-associated apolipoprotein genes in the brain are lacking. This review summarizes the rhythmic expression of clock genes and lipid metabolism-associated apolipoprotein genes within the SCN in *Mus musculus*. Nine of the twenty apolipoprotein genes identified from searching the published database (SCNseq and CircaDB) are highly expressed in the SCN. Most apolipoprotein genes (ApoE, ApoC1, apoA1, ApoH, ApoM, and Cln) show rhythmic expression in the brain in mice and thus might be regulated by the master clock. Therefore, this review summarizes studies on lipid-associated apolipoprotein genes in the SCN and other brain locations, to understand how apolipoproteins associated with perturbed cerebral lipid metabolism cause multiple brain diseases and disorders. This review describes recent advancements in research, explores current questions, and identifies directions for future research.

## 1. Introduction

Sleep is crucial for good health, whereas sleep deprivation affects learning, memory, attention, and physical health. Many factors, including sleep apnea, restless leg syndrome, insomnia, jet lag, shift work, and perturbation of the body’s internal clocks, can cause sleep disorders. Sleep disorders and sleep deficiency are strongly linked to a growing number of health problems, including heart disease, stroke, high blood pressure, type 2 diabetes, obesity, depression, and cancer.

The circadian rhythm is a 24 h cycle in which the body adjusts physically and behaviorally to the light and dark phases throughout the day. When the eye receives light from the surrounding environment, the body controls various factors, including sleep/wake cycles, eating habits, body temperature, hormone release, heart activity, and blood pressure [1,2,3]. However, disruptions to the circadian rhythm commonly occur; examples include clock gene mutations, medication use, stress, aging, irregular work schedules, changes in time zones, and vision impairment [4]. Frequent disruptions to the internal clock are a risk factor for multiple diseases and disorders, including cancer, sleeping disorders (e.g., jet lag), atherosclerosis, obesity, diabetes, viral infections (e.g., COVID-19), and neurodegenerative diseases (e.g., Huntington’s disease and Alzheimer’s disease) [5,6,7,8,9,10,11,12].

Many factors, such as cell phones, microwave radiation, temperature, and food availability have been found to regulate human brain diseases, as recently reviewed elsewhere [13,14,15]. Animal and human studies have indicated that the circadian rhythm system regulates lipid metabolism, including the diurnal rhythm of lipid absorption, storage, and transport [12,16,17,18,19,20,21,22,23,24,25,26,27,28,29]. The mechanisms through which disruption of the circadian rhythm and circadian clock affects the rhythmic expression of lipid metabolism-associated apolipoprotein genes in various brain regions remain unclear.

To understand the association between circadian clock genes and apolipoproteins, this review focuses on the regulation and dysregulation of the circadian clock and apolipoproteins in the brain.

Apolipoproteins, major constituents of lipoproteins, function as lipid transporters by binding lipids and facilitating their secretion into the blood, lymph, and cerebrospinal fluid (CSF). Apolipoprotein synthesis is affected by various factors, such as age and sex, through mechanisms that vary by tissue type. Clinical studies have shown that plasma levels of most apolipoproteins, except apolipoprotein (Apo)H, are higher in women than in men [30]. Regarding age, women show significant negative relationships with plasma levels of ApoB, ApoE, ApoH, and ApoJ from young to mid-life, whereas links at older ages are insignificant or positively related. Plasma levels of apolipoproteins including ApoA1, ApoA2, ApoB, ApoC3, and ApoH are negatively associated with age in middle-aged and old men; however, in men, plasma levels of ApoE and ApoJ show U-shaped curves with the period from young-aged to middle-aged, reacting to higher levels of ApoE and ApoJ in plasma at the oldest ages in men [30].

Many physiological functions and disease pathologies have been ascribed to apolipoproteins. For example, our animal studies have shown that the circadian clock regulates the ApoB-containing lipoprotein pathway, thereby controlling lipid absorption and plasma lipids, in clock circadian regulator (Clock)-mutant mice [31,32]. Global or liver-specific basic helix-loop-helix ARNT like 1 (Bmal1)-deficient mice show elevated assembly and secretion of an ApoB-containing very low-density lipoprotein (VLDL), thus regulating the development of atherosclerosis [26]. In addition, we have identified that ApoA-IV is targeted primarily by Bmal1, which down-regulates nuclear receptor subfamily 1 group D member 1 (Nr1d1), thereby controlling VLDL lipoprotein assembly and secretion in the liver via cyclic adenosine monophosphate (cAMP)-responsive element-binding protein H [33]. This process directly regulates ApoA4 expression in the liver and small intestine and consequently increases VLDL lipoprotein particle sizes in the blood circulation [33]. ApoA4, a crucial player in the circadian regulation of intestinal lipid absorption, mediates lipoprotein production [34]. A high concentration of total cholesterol in the serum in middle age is a risk factor for Alzheimer’s disease (AD) and other types of dementia in old age. Recently, studies have suggested a strong association between AD and cardiovascular disease (CVD) risk factors such as high-density lipoprotein (HDL) levels, low-density lipoprotein (LDL) levels, hypertension, and atherosclerosis [35]. Studies have shown that cholesterol-lowering agents such as statins decrease the incidence of AD [36,37], although the mechanism remains unknown.

In mammalian peripheral tissues, many genes associated with lipid biosynthesis and metabolism are rhythmically controlled by circadian clock genes [6,38,39,40], thereby suggesting that dysfunction in clock genes may be associated with abnormal lipid metabolism and impaired lipid absorption. For example, Nocturne (gene name *Ccrn4l*) plays a critical role in dealing with dietary lipids in the small intestinal enterocytes and, by extension, efficient absorption of lipids [19]; *Nocturne^−/−^* mice have diminished chylomicron/VLDL triglyceride and cholesterol levels [19]. Several studies have indicated that circadian clock regulation is necessary for normal physiology and pathology mechanisms.

The circadian clock may significantly regulate cerebral lipid metabolism and help prevent apolipoprotein-associated neurodegenerative diseases, such as AD [18,41,42,43,44,45]. Several apolipoproteins have been directly implicated in the etiopathology of AD, including APOE, clusterin (CLU or APOJ), APOC1, and APOB [46,47,48,49].

Here, we first explore the suprachiasmatic nucleus (SCN), the circadian clock, and apolipoproteins in the SCN; we then discuss the recently determined or assumed roles of apolipoproteins in brain physiology and diseases. This review does not cover all apolipoproteins’ functions and their modulation of lipid metabolism pathways or those involved in the various disease conditions of apolipoproteins; this information has been described in other reviews [49,50,51,52].

## 2. Function of SCN

### 2.1. SCN

The brain SCN is a minor area within the brain’s hypothalamus. One unilateral SCN of the brain contains approximately 10,000 neurons in two anatomic subdivisions. The SCN is the primary input and output location for light, as well as neuronal and hormonal activities [53,54,55]. The SCN includes many cell types, and many neurons contain multiple neuropeptides, neurotransmitters, and input or output connections. Wen et al. have identified eight primary cell types, each with a specific mode of circadian gene expression, according to single-cell RNA-sequencing [56]. Interestingly, several studies have shown that astrocytes execute the circadian rhythm in the SCN [57,58,59,60]. Studies have suggested that the SCN is the most critical region for sleep, mood, control of circadian timing, and non-neural circadian gene expression and regulation. Moreover, in mammals, removal of the SCN ablates the circadian rhythm [61,62,63], thus suggesting that the SCN is the primary center for circadian regulation. In addition, individual neurons of the SCN produce individualistic circadian oscillations of circadian clock gene expression and nerve reflex on the basis of ex vivo culture.

### 2.2. Neurons and Hormones in the SCN

Several peptide hormones, such as vasopressin (AVP), vasoactive intestinal peptide (VIP), peptide histidine-isoleucine, and neurotransmitters, are found in the SCN [64]. In the mouse, VIP is found in 10% of all SCN cells, whereas AVP neurons are found in 20% of all SCN cells [65]. AVP regulates blood pressure [66,67], whereas VIP governs the release of various bodily chemicals, such as gastric acid, during digestion [68]. Peptide histidine-isoleucine regulates food consumption behavior [69]. These hormones are essential in controlling energy and glucose homeostasis. The circadian rhythms of secretion of AVP and VIP have been found to be affected by gap junction (cell–cell junction) blockers in rat SCN slice cultures [70]. Because the removal of these gap junction blockers from cultures results in the restoration of the circadian rhythms of AVP and VIP, these hormones may be regulated by cell–cell interactions in the SCN, such as neuron-astrocyte cross-talk [70]. The central circadian clock may regulate physiological and pathological functions in peripheral tissues through hormones and peripheral tissue cross-talk.

Sex hormone receptors, such as estrogen receptor-α (ERα), estrogen receptor-β (ERβ), progesterone receptors, and androgen receptor (AR), are expressed in the SCN [71]. The SCN regulates the circadian rhythms of sex hormone release; conversely, sex hormone feedback influences SCN function. Several groups have reported that sex hormone receptors are associated with SCN function and the circadian clock in ERα-deficient and circadian clock gene knockout mice [72,73]. The difference in the SCN function between females and males has been suggested to be due to ERα expression, which is statistically significantly higher in the SCN in female than male animals [72]. Pre-astroglia (astrocytes and ependymal glia) have a sex-specific role in the organization of the SCN but not the sexually dimorphic area, and neuroglia have also been found to have a sex-specific role in the mature SCN in Mongolian gerbils [74].

Glucagon-like peptide 1 (GLP-1) is produced primarily in the intestine but is also found in the brain, particularly in the nucleus tractus solitarius, which is situated in the brainstem [75]. In addition, gene expression of GLP-1 is found in hypothalamus neurons, particularly in the paraventricular and arcuate nucleus [76]. The GLP-1 receptor is also expressed in the central and peripheral nervous systems. The FDA has recently approved synthetic GLP-1 receptor agonists for treating type 2 diabetes mellitus and weight loss [77,78]. Several studies in animal models have shown that GLP-1 receptor agonists may ameliorate motor and cognitive symptoms in Parkinson’s disease (PD) or AD [64], thus slowing the development of neurodegeneration.

### 2.3. Immune Factors in the SCN

The SCN has immense biological importance, functioning as the brain’s internal clock and guiding sleep patterns and ovulation cycles, as well as other regulatory functions. In the SCN, glial fibrillary acidic protein (GFAP), a specific marker for astrocytes, is present, and the levels of blood GFAP can be used to predict early-stage AD [79]. The glial cells of the SCN regulate the signals entering the circadian system by receiving them from the immune system via NF-kappaB signaling, according to studies in animal models [79,80]. Interleukin-1β (IL-1β) and interleukin-1 type 1 receptor (IL-1R1) are expressed in the SCN in young and old mice: IL-1β is more highly expressed in younger mice, whereas IL-1R1 shows rhythmic expression in only younger mice. The rhythmic phrases of both IL-1β and IL-1R1 in the paraventricular nucleus of the brain are affected by age in mice [81]. After treatment of mice with lipopolysaccharide (LPS; 5 mg/kg) to stimulate an immune response, only the expression of IL-1R1 increases after 6 or 24 h [81]. These data suggest that the levels of IL-1β and IL-1R1 in the mouse SCN are dependent on the diurnal clock, age, and the immune system. In animal models, LPS induces interferon-γ and tumor necrosis factor-α (TNF-α), thereby decreasing excitatory SCN activity [82]. Immune system dysfunction disrupts SCN function and can result in destructive sleep patterns. Thus, alterations in the synaptic mechanisms of SCN neurons might have integral roles in sleep disorders, and proinflammatory cytokines participate in these changes.

## 3. Circadian Rhythm of Circadian Clock Genes in the SCN

The SCN controls the central circadian clock, whereas peripheral clocks are located in every tissue and organ system of the body. In the brain, the two nuclei are located near the hypothalamus, and approximately 10,000 neurons hold a spectrum of changes occurring within the body activity throughout the day. Numerous circadian clock genes control the circadian rhythm, including *Clock*, *Bmal1*, period circadian regulators (*Per1*, *Per2*, and *Per3*), and cryptochrome circadian regulators (*Cry1* and *Cry2*). The *Bmal1* and *Clock* genes encode proteins that form a heterodimer. The heterodimer of Clock and Bmal1 binds sites called E-box enhancers and subsequently increases transcription upstream of the *Per1*, *Per2*, *Per3*, *Cry1*, and *Cry2* genes. After these genes are transcribed, the Per and Cry proteins heterodimerize, thereby preventing their self-transcription through interaction with the Bmal1:Clock/neuronal PAS domain protein 2 (Npas2) complex [83].

Several studies have reported that the circadian rhythm in humans is associated with neurodegenerative diseases; however, the mechanisms are unknown [84,85]. Kress et al. have shown that mice with peripheral Bmal1 deficiency have elevated ApoE in the brain parenchyma, which regulates the fibrillar plaque deposition [86]. Recently, Lee et al. have determined that Rev-erbα deficiency in microglia enhances lipid accumulation in the microglia and influences inflammatory signaling in male mice [28,87], thus suggesting that the circadian clock gene Rev-erbα might serve as a treatment target for AD.

**Figure 1:**
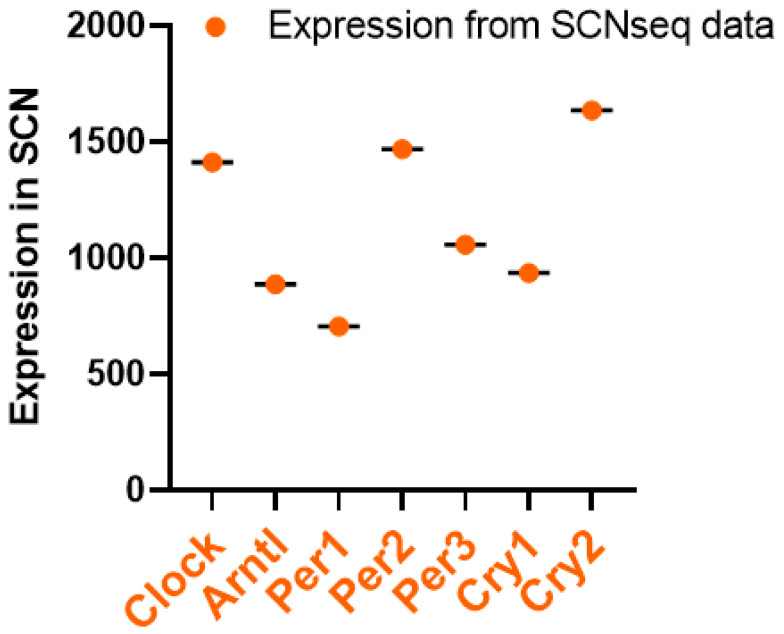
Circadian clock pathway gene expression levels in the SCN; data retrieved from SCNseq. Gene information, including expression levels, was obtained through the SCNseq database. The expression levels in the SCN were organized, and highly or weakly expressed genes were analyzed in GraphPad Prism 9.

**Figure 2:**
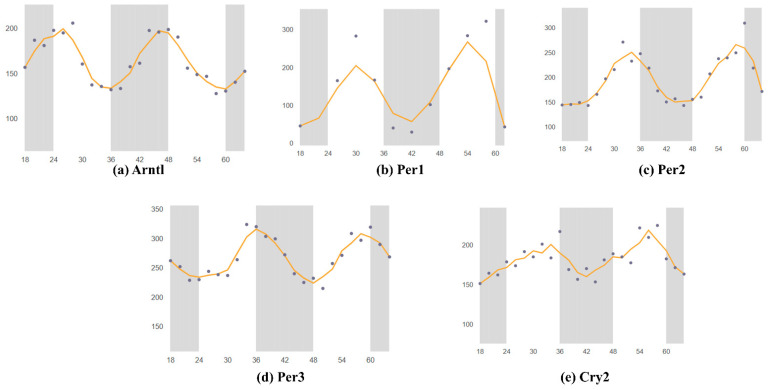
Mouse gene expression levels in the brain over two 24 h periods; data retrieved from CircaDB. The gray bar is dark period, dots is shown the different time gene expression through 66 h. Horizontal is over two 24 h periods 0–66 h. the wave pattern shown the circadian rhythm of the different time point data. (**a**) Arntl (Bmal1) expression in the brain stem; Mouse 1. OST Brain Stem (Affymetrix). (**b**) Per1 expression in the SCN; Mouse SCN MAS4 Panda 2002 (Affymetrix). (**c**) Per2 expression in the SCN; Mouse 1. OST SCN 2014 (Affymetrix). (**d**) Per3 expression in the brain stem; Mouse 1. OST (Affymetrix). (**e**) Cry2 expression in the SCN; Mouse 1. OST SCN 2014 (Affymetrix). Through the same database as in Figure 1, we determined whether each gene is rhythmically expressed over 24 h. The graphs are divided into two sections: light and dark, representing daytime and nighttime, respectively. The graphs were compared to determine when the genes were most highly expressed throughout the day. Similar patterns were identified in circadian rhythms. Past studies on Mus musculus brain tissue were collected through the Circadian Expression Profiles Database (CircaDB), compared with the graphs from SCNseq, and verified for consistency. The graphs from CircaDB also show the available data on the circadian rhythmic expression of the genes. Similar to the graphs from SCNseq, light and night phases are shown in the CircaDB graphs. High and low peaks were identified. All collected data from Affymetrix assays were searched, thus enabling analysis of gene expression levels.

Using the SCNseq database, we found that circadian rhythm-associated circadian clock genes are expressed in the SCN in male mice. Figure 1 shows a similar expression of all seven circadian clock genes, but the highest expression for Cry2. All identified circadian clock-associated genes show rhythmic expression over a 24 h cycle. The shaded parts of the graph represent the dark phases of the 24 h cycle. Similar patterns are shown in Figure 2. Bmal1 (*Arntl*), *Per1*, *Per2*, *Per3*, and *Cry2* all peak during the daytime and have the lowest expression in the nighttime. Furthermore, because expression levels were measured in mice, nocturnal behavior must also be considered: mice have an inactive phase during the daytime and an active phase during the nighttime.

As described above, the circadian cock regulates the immune system, which in turn plays a major role in the expression of circadian clock genes in the SCN. Adults with prenatal LPS exposure have elevated anxiety-like behavior along with altered rhythms of circadian clock genes in the SCN [88]: the most significant effect is on Nr1d1 expression, whereas Per2 is the least affected. However, the developing SCN also shows adaptive flexibility to immune challenges in the early stages of development. In the same study, whereas Nr1d1 expression in the LPS-treated animal group was arrhythmic at postnatal day 3, it showed improvements, on the basis of a higher amplitude, by postnatal day 20 [88], thus suggesting the circadian clock may assist the immune system in dealing with challenges later in life.

Deletion of Bmal1 in the SCN of the mouse brain leads to amyloid-β buildup within the brain. Moreover, disruption of the circadian rhythm of Bmal1 locally in the brain parenchyma increases the expression of ApoE, and fibrillar plaques begin to accumulate [89,90,91], thus suggesting that the circadian clock has an essential role in regulating amyloid-β and ApoE dynamics and pathology. AD is characterized by plaque accumulation in the brain, including amyloid-β buildup, and changes in the sleep cycle are symptoms of AD.

## 4. Apolipoproteins in the SCN and CSF

**Figure 3:**
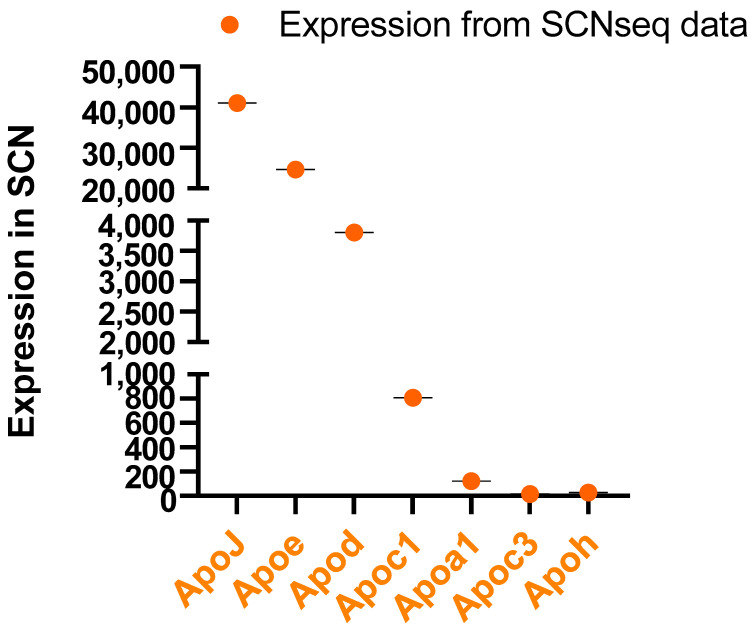
Apolipoprotein gene expression levels in the SCN; data retrieved from SCN seq, according to the methods in Figure 1.

As described above, the mouse circadian clock functions in regulating lipid synthesis and transport. Our group and several others have shown that circadian clock genes regulate lipid metabolism in peripheral tissues, such as the intestine and liver [12,16,17,20,39,92,93]. Apolipoproteins are essential in regulating tissue and plasma lipid levels in peripheral tissues and the central brain [86,94]. Lipoprotein particles contain multiple apolipoproteins. Apolipoproteins bind cholesterol, triglycerides, other lipids, and proteins and are grouped to become lipoproteins. Lipoproteins transport lipids among organs in the body’s circulation [95]. Specific apolipoprotein genes encode these apolipoproteins; specifically, in mammals, apolipoprotein E (*ApoE*), clusterin (*Clu*), apolipoprotein A1 (*ApoA1*), apolipoprotein C1 (*ApoC1*), apolipoprotein H (*ApoH*), and apolipoprotein M (*ApoM*) have been closely examined. Of the 22 known apolipoproteins, 9 are present in the central nervous system (CNS), and mRNAs of 8 apolipoproteins are found in the brain (Figure 3). Twenty years ago, Stuerenburg et al. determined that several apolipoproteins are present in 13–20 nm particles of the lipoprotein fraction; these apolipoproteins include ApoE, ApoA-I, ApoA-IV, ApoD, ApoH, and ApoJ [96]. ApoE is usually present in the largest lipoprotein particles; ApoA-I and ApoA-II are found in smaller lipoprotein particles; and ApoJ is present across the particle size range. Stuerenburg et al. have also found that ApoA-IV, ApoD, and ApoH are present in the CSF [96]. ApoE and ApoA1 are found in high concentrations in the CSF, in contrast to ApoJ, ApoD, ApoA-II, and ApoA-IV [96,97].

HDL, ApoAI, ApoJ, free fatty acids, and ceramides can cross the blood–brain barrier (BBB), whereas ApoE cannot [98]. Cholesterol cannot cross the BBB, whereas other cholesterol forms, such as 24-hydroxycholesterol (HC) and 27-HC, can do so (31698548). Lipids also interact with the BBB and affect its function. For example, HDL protects the BBB in multiple sclerosis, cholesterol may interfere with the BBB, and triglycerides suppress the transportation of leptin across the BBB [99].

Cholesterol is relevant to neurological issues, and cholesterol metabolism is controlled primarily by astrocytic activity in the brain. Adult neurons import cholesterol through ApoE-abundant particles via astrocytes instead of performing cholesterol biosynthesis. Cholesterol is necessary for a multitude of neural functions. Impaired cholesterol homeostasis affects amyotrophic lateral sclerosis, a common neurodegenerative disease in adults [100]. Neurons regulate cholesterol synthesis and metabolism through ApoE; cholesterol is then secreted from glial cells, specifically astrocytes. In the U373 (glioblastoma astrocytoma) astrocyte-derived cell line, cholesterol metabolism-associated protein expression is induced when glial cells are treated with nerve growth factors (NGFs). NGFs increase ApoE secretion and cholesterol concentrations [101]. Importantly, neuroprotection is suppressed when N1E-115 (an adrenergic cell line isolated from neuroblastoma C1300 in mice) cells are cultured with ApoE-silenced cells. NGFs are also necessary to activate neuroprotection in N1E-115 neurons [101].

Lipoproteins transport and process lipophilic substances within aqueous body fluids and regulate the immune system [102]. Relatedly, neurons are susceptible to immune responses and changes in the supply of lipids, thus further supporting a potential relationship between lipoproteins and neurodegenerative diseases.

HDL and plasma concentrations of certain apolipoproteins are potential biomarkers of covert brain infarcts. Koch et al. have found that in subspecies with low ApoC3, ApoJ, or ApoE concentrations of plasma, higher ApoA1 concentrations of plasma are correlated with fewer brain infarcts [103]. Similarly, in subspecies with low concentrations of ApoC3 or ApoJ, higher ApoE concentrations are associated with more covert infarcts [103]. Thus, HDL may potentially be used for drug delivery to tumors across the BBB, specifically for poorly soluble or unstable therapeutic agents [104].

Chua et al. have used targeted lipidomics to investigate the circadian pattern of lipids in the human brain [23]. In human studies, the authors have found that lipid metabolism disorders strongly influence circadian clock disruption [23]. Approximately 13% of healthy individuals’ plasma lipids show circadian rhythms [23], thus suggesting that metabolic disturbances may be associated with circadian rhythm disruption.

Decades of human and animal research have demonstrated that apolipoproteins and the SCN system interact at levels of biological function as diverse as molecular-level gene expression and multiple behaviors. However, little is known regarding the mechanisms and roles of apolipoproteins in the SCN, and no studies have investigated the circadian rhythm of apolipoprotein regulation in this brain region.

## 5. Circadian Rhythm of Apolipoprotein Genes in Mouse SCN

**Figure 4:**
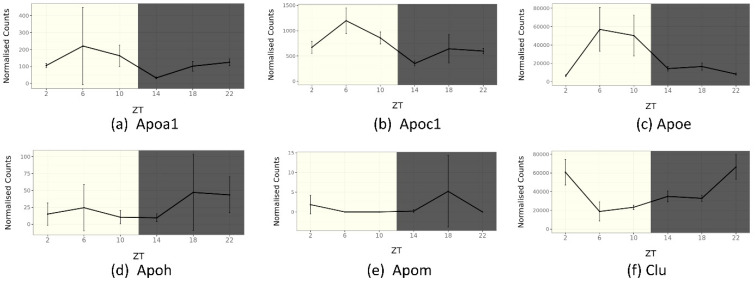
Mouse gene expression levels over 24 h in the brain; data retrieved from SCNseq. The yellow color is light period, dark bar is night period (no light). ZT: Zeitgeber Time. Horizontal is ZT from 0–24 h. Mean ± SD. (**a**) Apoa1 expression levels. (**b**) Apoc1 expression levels. (**c**) Apoe expression levels. (**d**) Apoh expression levels. (**e**) Apom expression levels. (**f**) Apoj/Clu expression levels. Methods as described in Figure 2.

Apolipoproteins are essential for controlling lipid metabolism in the brain. We verified the house mouse (*Mus musculus*) apolipoprotein genes from the SCNseq and CircaDB databases to understand how lipid metabolism perturbations might be associated with circadian rhythm disruption. ApoJ (Clu) has higher expression in the SCN than ApoE and ApoC1 (Figure 3). Therefore, these apolipoproteins appear to be expressed and synthesized within the brain tissue. Some apolipoproteins have been described in brain locations other than in the SCN. Apolipoprotein genes such as ApoC3, ApoH, and ApoM, are weakly expressed in the SCN and therefore might be synthesized in peripheral tissues and brought to the brain through the circulation. However, expression of ApoB and ApoA4 has not been observed in the SCN in mice (Figure 3). Interestingly, ApoA1, ApoC3, and ApoH are also expressed in the SCN. In addition, several apolipoprotein genes show rhythmic expression (Figure 4). The expression of ApoM has not been observed in the SCN, according to the SCNseq database, but changes in expression of ApoM have been observed over a 24 h cycle in the overall cerebral tissue, according to the CircaDB database. ApoA1, ApoC1, and ApoE all peak in the daytime, when mice are inactive. In contrast, ApoH, ApoM, and Clu show the greatest increase in activity during the active phase.

Several apolipoprotein genes, such as the *ApoE* and *Clu* genes, exist in the brain and have a known relationship to AD. We have not reviewed all apolipoproteins that may bind/transport lipids; instead, we focus on apolipoprotein genes that have been studied in recent years and detected in the SCN at the gene expression level through SCNseq. (http://www.wgpembroke.com/shiny/SCNseq/, in 20 July 2022). We will further explain how these genes contribute to the risk of brain diseases, including AD.

### 5.1. ApoA1

ApoA1 (ApoA-I) is synthesized in brain endothelial cells and presented in the CNS pool [105,106]. ApoA1 protein is found in the CSF, and ApoA1 mRNA is not detected in brain tissue. ApoA1 is a central component of HDL particles, which transport cholesterol from peripheral tissues to the liver. Despite being composited in the liver, intestine, and kidneys, ApoA1 can enter the brain at the choroid plexus, crossing the BBB. Therefore ApoA1 has been implicated in the brain lipid homeostasis [107]. ApoA1 has not been explored in depth in the development of AD. As a major component of HDL, this apolipoprotein is involved in transporting cholesterol from peripheral tissues to the liver.

ApoA1 is linked to a variety of neurodegenerative diseases, such as PD and schizophrenia [108]. When ApoA1 enters the brain, it has been found to colocalize with Amyloid beta (Aβ) plaques in the cortex in patients with AD [102], thus suggesting that ApoA1 might affect amyloid precursor protein (APP) cleavage, Aβ aggregation, and Aβ clearance. Vollbach et al. have shown that human polymorphisms in the promoter region of the APOA1 gene (−75 A/G) are associated with early AD pathogenesis; individuals homozygous for the A allele of ApoA1 develop AD approximately 8 years faster than heterozygotes [109,110].

### 5.2. ApoA4

Apolipoprotein A4 (also known as ApoA-IV) is a plasma protein that is found primarily in HDL and on the surfaces of newly synthesized chylomicrons and controls lipid absorption, plasma lipids, and transport of cholesterol in lipoproteins from peripheral cells back to the liver via several steps of the reverse cholesterol transport pathway [33,34,111].

ApoA-IV is produced in the hypothalamus and colocalizes with neurons and glia in rat brain regions [112,113]. It is also detected in astrocytes of the mouse brain [114]. A high-fat meal enhances the expression of ApoA-IV in various organs, and ApoA-IV has been found to play a central role in satiety in animal models [115,116,117]. In human studies, a polymorphic version of ApoA-IV has been shown to highly efficiently activate lecithin cholesterol acyl transferase, and a polymorphic version of ApoA-IV may be linked to high AD risk [118]. In APP/presenilin 1 (PS1) transgenic mouse model studies, genetic ablation of *APOA4* has been suggested to promote AD pathogenesis [119]. In human studies, the *APOA4* codon 360 mutation (C > T) has been associated with high AD risk [120,121]. However, the exact mechanism through which ApoA4 influences AD and, subsequently, lipid metabolism in the brain remains unclear.

### 5.3. ApoB

ApoB in the liver is controlled by several factors such as hormones (e.g., insulin), dietary factors (fatty acid), and varying therapeutic drugs (e.g., statins, niacin, and fibric acids) [122]. As described above, cerebral ApoB mRNA is not detected with RNA-Seq techniques. However, ApoB-containing lipoproteins are observed in the CSF. Pre-symptomatic individuals have been shown to have elevated apoB, as well as t-tau, p-tau, and the aforementioned synaptic markers, in the CSF. Thus, human ApoB levels in CSF correlate with early tau dysregulation and may aid in identifying individuals predisposed to the development of visuospatial cognitive decline [96,123]. Thus, ApoB is an essential marker of initial tau pathology in AD. Further studies are necessary to fully understand the mechanism underlying the link between ApoB and AD in animals or humans.

### 5.4. ApoD

ApoD is detected in brain regions including the SCN, cortex, and cerebellum [124]. Cerebral ApoD protein is synthesized and excreted from the brain mainly by oligodendrocytes and astrocytes and secreted as lipoproteins. ApoD has gained attention for its expression in various disease conditions and its wide-ranging presence in tissues and subcellular locations. ApoD activity is associated with lipid regulation rather than lipid transport [124]. Additionally, ApoD regulates various health and disease processes by controlling the redox states of lipid structures [125]. In normal conditions, neuronal ApoD protein expression is observed in Purkinje neurons and, to a lesser extent, in cortical neurons. In pathological conditions, ApoD protein may increase in the CSF [126] and affect different brain regions [127,128,129], although the underlying mechanisms are not fully understood.

### 5.5. ApoE

-Human APOE-

Human ApoE has three main alleles, ApoE2, ApoE3, and ApoE4. The presence of variants of the human APOE gene may explain the links among microglial cells, synaptic dysfunction, and lipid metabolism dysfunction [130]. More than 20% of the population carries the ApoE4 gene, and 40% of people with the ApoE4 gene are obese. Consequently, people with ApoE4 variants may be at elevated risk of neurodegenerative diseases such as AD, late-stage AD, Lewy body dementia, depression, traumatic brain injury (TBI), stroke, and spinal cord injury (SCI).

Three human ApoE alleles and six phenotypes exist, each with different metabolic implications and plasma cholesterol levels. Defective ApoE2 is associated with type III dysbetalipoproteinemia due to inefficient clearance of triglyceride-rich lipoproteins. Research has focused on deciphering the function of ApoE in neural β-amyloid deposition to understand why ApoE4 is a risk factor for Lewy body dementia and AD [131]. Recently, studies using human iPSC-derived cerebral organoid models have found that a lack of cerebral ApoE expression increases α-synuclein and lipid buildup—characteristics of AD and dementia. Greater α-synuclein buildup has been observed in ApoE4 carriers than in ApoE3 carriers [132]. The expression of ApoE2 and ApoE3 has been found to prevent lipid accumulation.

As previously described, ApoE4 is a well-known risk factor for AD development, specifically that of late-stage AD. Recently, Pohlkamp et al. have reported that phenotypes such as impaired endolysosomal trafficking, breakdown of synaptic homeostasis, and decreased amyloid clearance are due to the isoelectric point of ApoE4 matching the pH of the early endosome, thus delaying dissociation from ApoE receptors [133]. Furthermore, Montagne et al. have shown that disruption of the BBB promotes ApoE4-associated cognitive decline [134].

To study the role of human *ApoE* polymorphisms in atherosclerosis, lipid metabolism, and AD at the animal level, humanized mouse models substituting ApoE4 for the mouse *ApoE* gene have been developed [135]. ApoE4 causes neuronal hyperexcitability in knock-in mice susceptible to AD; ApoE4 carrier status has been linked to symptoms of depression [136]. Although data confirming an association between diminished cognitive ability and depression are lacking, depression may be associated with ApoE status and hippocampal volume but not cognitive decline in aging adults with TBI [137]. ApoE levels in the amygdala and prefrontal cortex have been found to predict relative regional brain volumes in irradiated Rhesus macaques [138]. The disease risk caused by ApoE4 also depends on its effects on β-amyloid deposition; regardless of diagnosis, the gene is associated with elevated β-amyloid levels [131].

APOE status is associated with dysfunction in inflammatory pathways in AD. More neurological pathways are impaired in ApoE4 carriers with AD than in ApoE3 carriers [139]. ApoE4 mice develop a leaky BBB [140], increased matrix metallopeptidase 9 (MMP9), damaged tight junctions, and decreased astrocyte end-foot coverage of blood vessels, whereas ApoE2 and ApoE3 mice do not show effects on MMP9 [140]. These responses are ameliorated by the removal of ApoE4, whereas the removal of ApoE3 does not affect BBB integrity; therefore, ApoE4 is one of the main reasons for BBB dysfunction; consequently, the alleles of ApoE4 in astrocytic production are responsible for regulating its integrity [140].

APOE4 overexpressing mice have higher ceremide levels in the cortex than ApoE3-overexpressing mice. Moreover, a mouse model of familial AD has shown elevated ceremide levels in the cortex. Older mice (>5 months of age) show higher sphingosine-1-phosphate (S1P) levels in three brain regions than younger mice (<3 months of age). Ceramide levels are lower in the hippocampus but higher in the cortex in female mice than in male mice with ApoE4 overexpression [141]. Thus, sex, as compared with ApoE genotype, familial AD history, and age, substantially influences neural ceramide levels in mice.

ApoE4 can be produced by neurons when the brain is under stress, such as during aging. In in vitro cultured neuron models from various cellular sources, including astrocytes and neurons, ApoE isoforms have shown differing effects on neural activity in controls and AD-like models [141]. While astrocyte-expressed ApoE4 increases neuronal activity, neuron-expressed ApoE induces a higher neuronal firing rate in humanized ApoE3 than in humanized ApoE4. Studies have suggested that ApoE has other implications and consequences on neuronal activity depending on whether it is generated by astrocytes or both neurons and astrocytes. Human ApoE4 induces more robust neuronal firing in astrocytes, whereas ApoE3 promotes greater neuronal activity.

ApoE3 overexpressing mice show a neural response to a high-fat diet, whereas findings in ApoE4 overexpressing mice do not support a model wherein an early inflammation imbalance in ApoE4 brains precipitates increased brain CNS damage [142]. Sortilin is a receptor that works with ApoE in stimulating the polyunsaturated fatty acids into becoming neuromodulators and creating anti-inflammatory genes within the brain, thus providing cerebral protection. This receptor is present in ApoE3 carriers but absent in ApoE4 carriers, thus suggesting that perturbed cerebral lipid metabolism is a characteristic of AD in ApoE4 carriers [143].

Many studies have reported specific interactions or correlations between the expression of human ApoE genes in the brain and their known associations with AD; however, whether the rhythm of the ApoE gene in the brain is associated with AD remains unknown.

-Mouse ApoE-

Mouse ApoE has only one isoform. ApoE is a lipid-transport protein known to control the inflammatory characteristics of activated microglia in various neurodegenerative diseases. Microglia are activated early in prion pathogenesis, resulting in microglia-driven neuroinflammation and deterioration of neural networks. The infection of control wild-type mice with 22 L prions has been found to increase ApoE expression in activated microglia but reduced expression of triggering receptor expressed on myeloid cells 2. In mice, ApoE is required for adequate hippocampal and hippocampal neurogenesis function after TBI [144]. Female mice show a significant role in regarding alcohol consumption in the context of TBI condition. In addition, ablation of ApoE augments prion protein (PrP)-modulated neurodegeneration [145]. Moreover, ApoE isoforms have different effects on neuronal activity in APP/PS1 AD transgenic mice compared with wild-type mice [146].

ApoE-deficient (*Apoe^−/−^*) mice enable the study of the similarities between atherosclerosis models and human pathology and diet [147]. *Apoe^−/−^* mice have short-term disease incubation periods, as well as enhanced spongiform lesions, neurodegeneration, astrocytes, and microgliosis. *Apoe^−/−^* mice show enhanced neurotoxic A1 reactive astrocytes, and microglia show increases in markers indicative of a microglial neurodegenerative phenotype. *Apoe^−/−^* mice show diminished clearance of normal cellular PrP and damage neurons in microglia; thus, excess debris leads to neuroinflammation and neuronal death [145].

A significant link exists between mouse brain ApoE and Aβ production in vivo, and ApoE has been found to regulate Aβ synthesis by in vitro system studies [148]. Wang et al. have shown that astrocyte ApoE transports cholesterol from astrocytes to neurons; ApoE regulates and interacts with APP and synthesizes Aβ peptide in neurons. Knockdown of cholesterol synthesis genes or treatment of astrocytes with cholesterol-free ApoE decreases cholesterol levels in cultured neurons and promotes APP trafficking to lipid clusters. While the amount of cholesterol within the neurons increased, increasing the product of APP interaction with β- and γ-secretases generated Ab peptide to develop AD. Furthermore, ApoE has been found to have many pathophysiological functions, such as oxidative stress, stabilization of neuronal microtubules, synaptic plasticity, apoptosis, and immunomodulation in animal model studies; these functions might have important roles in controlling the development of AD.

### 5.6. ApoC1

ApoC1 (ApoC-I) is expressed primarily in the liver and macrophages, but it is also expressed in the SCN. ApoC-I mRNA and protein expression have been observed in brain astrocytes, and they have been found to decrease with age [149,150,151]. Polymorphisms in the human ApoC1 gene are associated with altered transcription of ApoC1, thus increasing AD development, and blood total cholesterol and triglycerides [152,153]. ApoC1 has a central role in the metabolism of HDL and VLDL. According to animal studies, ApoC1 acts on lipoprotein receptors through several mechanisms, such as ApoC1 inhibiting the binding of ApoE-contained lipoprotein to their receptor; ApoC1 reducing the activity of lipoprotein lipase (LPL) and hepatic lipase and consequently enhancing blood triglyceride levels; decreasing phospholipase A2; and downregulating cholesteryl ester-transfer-protein and activating lecithin-cholesterol transferase, thus controlling blood cholesterol levels [52]. Several studies have shown that ApoC1 is linked to several pathways, including plasma lipoprotein assembly, remodeling, and clearance, and signaling by the liver X receptors LXRα and LXRβ [154,155,156]. ApoC1 has an important function in triglyceride-rich lipoproteins by inhibiting the binding of VLDL to the VLDL-receptor, LDL-receptor, and LDL-receptor-associated protein (LRP) and downregulating the activity of LPL.

Studies have shown that global ApoC1 knockout (*apoC1^−/−^*) mice have impaired hippocampal-dependent memory and elevated expression of TNF-α. Mice overexpressing human ApoC1 have impaired learning and memory. These studies have indicated that ApoC1 loss or gain of function decreases learning and memory [157]. ApoC-I prevents the ApoE–receptor interaction; furthermore, the ApoE4 allele usually causes enhanced expression of ApoC1 [158]. Several functions of ApoC1-associated lipid metabolism in the brain play essential roles in CVD. Future research must determine whether the rhythmic expression of ApoC1 is associated with brain lipid metabolism and AD.

### 5.7. ApoH

ApoH is a phospholipid-binding (e.g., cardiolipin) plasma protein expressed in astrocytes and neurons of the brain. ApoH synthesis occurs in the brain. ApoH proteins have been observed in the CNS and CSF; ApoH, like ApoA4, can enter the brain in “lipid-poor” particles or as free apolipoproteins [96].

Human ApoH levels in the brain and plasma have been associated with cognitive aging, predementia syndrome, mild cognitive impairment, and AD in older individuals [159]. Therefore, studying ApoH is essential to better understand the factors affecting the extent of its effects on brain disease. Plasma ApoH levels are linked to cognitive status, whereas ApoH single nucleotide polymorphisms are associated with type 2 diabetes, chronic inflammatory disease, and age-associated cognitive performance [160]. In peripheral tissues, ApoH has been associated with physiological pathways such as lipoprotein metabolism, coagulation, and antiphospholipid autoantibody production. A genome-wide association study has shown that ApoH is a new locus associated with lipoprotein (a) levels [161]. In lupus and primary antiphospholipid syndrome patient samples, ApoH may be a cofactor required for anionic phospholipid binding [162].

### 5.8. ApoJ

Expression of ApoJ, also called Clusterin, has been observed in astrocytes, neurons, and the ependymal cells lining the ventricles of the brain [159]. ApoJ-containing lipoproteins such as HDL are generally small and contain few lipids. ApoJ plays an important role as an essential determinant of CSF cholesterol efflux capacity; ApoJ mitigates the risk of mild cognitive injury and AD via the cellular efflux of cholesterol or other lipids [159]. ApoJ acts as a molecular chaperone in response to cellular stress [102,163,164]. ApoJ binds Aβ fibrils and is correlated with Aβ plaques, neuropil threads, and cerebrovascular amyloid accumulation in the brain in AD [102,165,166]. ApoJ suppresses Aβ aggregation and controls Aβ transport across the BBB; thus, ApoJ may have neuroprotective effects. ApoJ has been identified to play an essential role in neurodegenerative conditions, such as Lewy bodies and prion deposition, and pathological conditions, such as AD, gliomas, TBI, multiple sclerosis, ischemia, epilepsy, SCI, aging, and chemically induced lesions [102,167,168]. ApoJ knock-out (ApoJ^−/−^) mice display significantly impaired recovery from cerebral ischemic insult, thus suggesting that ApoI protects against these injurious states.

### 5.9. ApoM

ApoM is included in HDL, LDL, and the triglyceride-rich lipoproteins VLDL and chylomicrons; all of these lipoproteins are involved in lipid transport. Single nucleotide polymorphisms of the ApoM gene are associated with several prevalent diseases, including T2DM, chronic obstructive pulmonary disease, and systemic lupus erythematosus [169,170,171]. The function and possible roles of ApoM in the brain and SCN are not understood. Studies have determined that ApoM is released by tubular epithelial cells and reabsorbed in a megalin-dependent manner [158]; however, ApoM is not found in the urine under normal physiological conditions in mouse models. Several studies have reported that ApoM-deficient mice have lower blood glucose levels than control wild-type mice, and ApoM enhances insulin secretion and sensitivity [169,172,173]. Janiurek et al. have shown that ApoM-bound S1P maintains low paracellular BBB permeability in all cerebral microvessels and decreases vesicle-associated transport via penetrating arterioles [174]. ApoM is essential for the formation of pre-β-HDL and for reverse cholesterol transport [175]. Interestingly, ApoM levels are lower in ApoA1 knockout mice than wild-type mice and thus may be associated with the pathogenesis of cardiometabolic diseases.

## 6. Apolipoproteins and Diseases

The SCN controls apolipoprotein and lipid metabolism-associated genes. Nine apolipoprotein genes show rhythmic expression in brain tissue, thus suggesting a connection between the circadian master clock and cerebral lipid metabolism. Studies have demonstrated that a disrupted circadian clock is a high-risk factor for brain diseases, specifically AD. The rhythmic expression of lipid metabolism-associated apolipoprotein genes confirms the association between AD and the SCN. Thus, disruptions to the circadian clock impair lipid metabolism. Additionally, ApoE and Clu show higher expression than other apolipoproteins in the brain and thus might be required within the brain to control brain function. ApoE is highly correlated with AD; ApoE4, a form of human ApoE, increases the risk of AD [176]. Therefore, future research must determine whether genes with similar rhythmic expression are associated with AD. Identifying the other genes that affect circadian rhythms through the use of conditional knockout mice will be essential. In addition, the mechanisms through which the circadian clock, particularly in the SCN, influences the expression of apolipoprotein genes, and subsequently lipid metabolism, remain unknown.

Scientists must include the circadian rhythm as a crucially important variable in experimental protocols. The roles of apolipoproteins in maintaining health, preventing brain disease, and treating illnesses will be increasingly clarified by research expanding understanding of the brain to SCN neurons and hormones, immune factors, and the circadian clock, all of which affect apolipoprotein expression. However, despite the different pathological effects of apolipoproteins, treatment goals for AD, atherosclerosis, and T2DM may be achieved.

## 7. Perspectives

**Figure 5:**
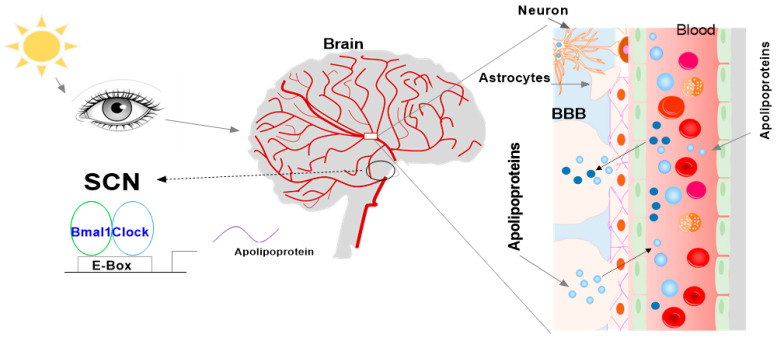
Two pathways of apolipoproteins are present in the brain. One hypothesis is that apolipoproteins are expressed and synthesized in the brain, are directly regulated by the central circadian clock, and are indirectly linked to the peripheral circadian clock. The second hypothesis is that apolipoproteins in the circulation enter the brain through the BBB; apolipoproteins may be directly associated with the peripheral circadian clock and indirectly associated with the central circadian clock. The two pathways may control physiological function and pathology in diseases.

Despite extensive studies in peripheral tissues of the body, further investigation of the circadian rhythm of the clock and apolipoproteins in the brain is necessary. Thus, this review described the expression levels of relevant genes in the SCN and whether they show rhythmic expression over a 24 h cycle in the brain in mice. We hypothesized that all identified genes crucial to the circadian clock and apolipoproteins are regulated by the SCN in the brain in mice and thus should show rhythmic expression, reacting to the light and dark phases over 24 h. We propose that two apolipoprotein pathways are present in the brain (Figure 5). The first hypothesis is that apolipoproteins are expressed and synthesized in the brain, are directly regulated by the central circadian clock, and are indirectly linked to the peripheral circadian clock. The second hypothesis is that circulating apolipoproteins enter the brain through the BBB; apolipoproteins may be directly associated with the peripheral circadian clock and indirectly associated with the central circadian clock. Apolipoproteins in these two pathways may control the apolipoprotein-associated physiological function and pathology in diseases.

To our knowledge, the functions of apolipoprotein signaling pathways in the SCN and other brain locations might be associated with several brain diseases and have not been completely elucidated under normal physiology. Further studies are necessary to thoroughly investigate the mechanisms of apolipoproteins in the pathogenesis of brain diseases and determine their ability to serve as novel therapeutic targets.

## Data Availability

Publicly available datasets were analyzed in this study. This data can be found here: [http://circadb.hogeneschlab.org/ (Pizarro, et al., 2013 [177]); http://www.wgpembroke.com/shiny/SCNseq/, Pembroke, et al., 2015 [178]; https://cgdb.biocuckoo.org/links.php].

## Abbreviations

AD: Alzheimer’s disease; Aβ: amyloid beta; Apo: apolipoprotein; APP: amyloid precursor protein; AR: androgen receptor; AVP: vasopressin; BBB: blood brain barrier; Bmal1: basic helix-loop-helix ARNT like 1 (ARNTL); cAMP: cathelicidin antimicrobial peptide; Clock: clock circadian regulator; Clu: clusterin or ApoJ; CNS: central nervous system; Cry: cryptochrome circadian regulator; CVD: cardiovascular disease; CSF: cerebrospinal fluid; ERα: estrogen receptor-α; ERβ: estrogen receptor-β; GFAP: glial fibrillary acidic protein; GLP-1: glucagon-like peptide 1; HDL: high-density lipoprotein; IL-1R1: interleukin-1 type 1 receptor; IL-1β: interleukin-1β; LDL: low density lipoprotein; LPS: lipopolysaccharide; Lpl: lipoprotein lipase; MMP9: matrix metallopeptidase 9; mRNA: messenger RNA; NGF: nerve growth factor; Nr1d1: nuclear receptor subfamily 1 group D member 1; PD: Parkinson’s disease; Per: period circadian regulators; PrP: prion protein; PS1: presenilin 1; S1P: sphingosine-1-phosphate; SCI: spinal cord injury; SCN: suprachiasmatic nucleus; TBI: traumatic brain injury; TNF-α: tumor necrosis factor-α; VIP: vasoactive intestinal peptide; VLDL: very low-density lipoprotein
